# Trends in hospital admission for asthma in Brazil 1998-2010

**DOI:** 10.1186/1939-4551-8-S1-A16

**Published:** 2015-04-08

**Authors:** Ivan Duarte, Gustavo Graudenz

**Affiliations:** 1 Uninove, Brazil; 2 Nove De Julho University, Brazil

## Background

Asthma is a worldwide disease, affecting about 300 million people over the world, with reports of increased prevalence the last decades. Asthma is the forth cause of hospitalizations in Brazil. Hospital admissions to achieve clinical control of asthma are responsible for a significant part of the direct costs on health care by the Brazil’s Unified Health System (SUS), and affect the quality of life of patients and their families. Recent advances in medical therapy and improvements in providing access to asthma control programs can lead to rapid changes in rates of hospital admission. This paper updates the trends in hospitalizations due to asthma in Brazil.

## Methods

This is a time series study. We used J45 and J46 code for asthma, according to International Classification of Diseases (ICD10), from 1998 to 2010. Initially, we calculated coefficients of asthma according to age groups, gender and location, using the demographic and morbidity data provided by Department of Informatics of the SUS. (DATASUS) Then, the coefficients were placed in a scatter diagram to visualize the function that best explains the temporal trend. After, models were fitted to choose equations that best explained the patterns observed.

## Results

All rates linearly decreased, showing a downward trend in the number of hospitalizations. Brazil experienced a reduction of 55.2% in the total hospitalization crude rates for asthma, in the period from 1998 to 2010, with a drop of 12.632 hospital admission per year per 100,000 inhabitants. The extremes of age showed the greatest annual reduction. There was a reduction of 40.192 events of hospitalization among children under four years old and a drop of 24.511 cases among the elderly over 75 years old, per 100.000 age-adjusted inhabitants. The Brazilian macro regions that showed greater decline were the Northeast, South and Midwest with a decrease between 18 to 16 region-adjusted hospital admissions per year. Women showed a decrease of 13.544 and men 11,626 annually gender-adjusted cases per 100,000 individuals, with convergent trend to men`s during the studied period.

## Conclusions

Despite the existing evidence of an increasing prevalence of the disease, there is a steady decline in hospital admissions due to asthma in Brazil during the observed period.

